# Alzheimer’s Prevention Lifestyle Risk Coaching in Low-income Older Adults

**DOI:** 10.1093/geroni/igab046.2755

**Published:** 2021-12-17

**Authors:** Faika Zanjani, Annie Rhodes

**Affiliations:** Virginia Commonwealth University, Richmond, Virginia, United States

## Abstract

Prevention, with widespread lifestyle risk reduction at the community-level, is currently considered an effective method to decrease Alzheimer’s disease (AD). As part of the Virginia Commonwealth University iCubed Health and Wellness in Aging Core, diverse older adults (60+) living in Richmond, VA, with incomes below $12,000/year and managing either diabetes/cardiovascular symptoms, were offered weekly lifestyle telephone-health coaching for 12-weeks, providing education, motivations, self-efficacy, and referral services for AD lifestyle risk. The study sample (n=40, mean age 68 years (range: 60-77 years) was 88% African American/Black (n=35), 100% Non-Hispanic, and 45% males (n=18)). Thirty-nine (95%) of subjects successfully participated in coaching sessions; on average 91.9% (11) sessions/subject were completed. On a scale of 0-100 (higher scores more positivity), rated their experience 93.3. On a scale of 0-10 (higher scores more improvement), rated their health improved 8.36. Pre/post-test analyses indicated lifestyle improvement trends over 4-months for total lifestyle risk (F=4.69;p=.037;effect=.12), social activity (F=4.63;p=.063;effect =.09), and improvement in certain psychological domains: AD knowledge (F=4.49;p=.041;effect=.11); cognitive functioning (short-term memory (F=4.99;p=.038;effect= .21); delayed memory (F=2.26;p=.154;effect=.11); Trails A (F=5.60;p=.0294;effect=.24); and Trails B (F=2.22;p=.154;effect=.11). Participants provided positive anecdotal feedback and the need for continued health coaching. In conclusion, this preliminary work creates the impetus for future large-scale lifestyle AD prevention investigations to improve the lives of AD-risk, low-income, diverse older adults. These findings demonstrate that telephone-based health coaching is feasible, based on participant engagement, and effective, based on positive trends in reducing AD risk factors.

